# Reactive Oxygen Species-Activated Nanoprodrug of Ibuprofen for Targeting Traumatic Brain Injury in Mice

**DOI:** 10.1371/journal.pone.0061819

**Published:** 2013-04-24

**Authors:** Morgan A. Clond, Bong-Seop Lee, Jeffrey J. Yu, Matthew B. Singer, Takayuki Amano, Alexander W. Lamb, Doniel Drazin, Babak Kateb, Eric J. Ley, John S. Yu

**Affiliations:** 1 Department of Surgery, Division of Trauma and Critical Care, Cedars-Sinai Medical Center, Los Angeles, California, United States of America; 2 Department of Neurosurgery, Cedars-Sinai Medical Center, Los Angeles, California, United States of America; University of South Florida, United States of America

## Abstract

Traumatic brain injury (TBI) is an enormous public health problem, with 1.7 million new cases of TBI recorded annually by the Centers for Disease Control. However, TBI has proven to be an extremely challenging condition to treat. Here, we apply a nanoprodrug strategy in a mouse model of TBI. The novel nanoprodrug contains a derivative of the nonsteroidal anti-inflammatory drug (NSAID) ibuprofen in an emulsion with the antioxidant α-tocopherol. The ibuprofen derivative, Ibu_2_TEG, contains a tetra ethylene glycol (TEG) spacer consisting of biodegradable ester bonds. The biodegradable ester bonds ensure that the prodrug molecules break down hydrolytically or enzymatically. The drug is labeled with the fluorescent reporter Cy5.5 using nonbiodegradable bonds to 1-octadecanethiol, allowing us to reliably track its accumulation in the brain after TBI. We delivered a moderate injury using a highly reproducible mouse model of closed-skull controlled cortical impact to the parietal region of the cortex, followed by an injection of the nanoprodrug at a dose of 0.2 mg per mouse. The blood brain barrier is known to exhibit increased permeability at the site of injury. We tested for accumulation of the fluorescent drug particles at the site of injury using confocal and bioluminescence imaging of whole brains and brain slices 36 hours after administration. We demonstrated that the drug does accumulate preferentially in the region of injured tissue, likely due to an enhanced permeability and retention (EPR) phenomenon. The use of a nanoprodrug approach to deliver therapeutics in TBI represents a promising potential therapeutic modality.

## Introduction

In industrialized countries, traumatic brain injury (TBI) is the leading cause of death in those under the age of 45 [1]–[3] and traumatic injuries account for a greater number of potential years of life lost than all other causes of death [4]. According to most recent CDC estimates (2004–2006), there are 1.7 million new cases of TBI annually, with 52,000 deaths, 275,000 hospitalizations, and 1.4 million people treated in emergency departments each year [5].

For both patients and society at large, traumatic brain injury carries a large cost burden estimated for the United States to be $60–100 billion per year due to resulting healthcare costs and lost productivity [6], [7]. For survivors, the impact of TBI on quality of life is significant, as most suffer some degree of cognitive impairment that may include memory or motor deficits, psychological disorders, sleep disturbances, or seizures, with an increased risk for developing neurodegenerative diseases or other encephalopathies later in life [8], [9]. Currently, an estimated 5.3 million people live in the US with a permanent TBI related disability [5].

Decades of clinical and basic science trials have attempted to improve outcomes of traumatic brain injury using a wide variety of novel treatment strategies. The most recent trials have investigated drugs such as calcium channel inhibitors [10]–[12], dexanabinol [13], [14], minocycline [15], [16] and magnesium [17], [18]. Unfortunately, no interventions have been successful enough in practice to be implemented as standard of care [19]–[21].

Among the animal models available for traumatic brain injury, the controlled cortical impact (CCI) method represents a refined and highly reproducible means of producing several gradations of injury [22]. CCI can be conducted as either a closed or open injury with or without burr hole exposure of the brain. The CCI model, first described in 1988 by Lighthall et al., allows for the control of multiple parameters of injury, including the velocity, duration, penetration depth, and contact area of the impact [23]. Other commonly used models include fluid percussion injury, first described in 1965 by Lindgren and Rinder [24], and weight drop described by Feeney et al in 1981 [25]. Very recently, mouse models for blast injury have been developed for the purpose of reproducing the common battlefield injury, and investigating the distinct pathologies associated with this mechanism of injury [26], [27].

CCI recapitulates the characteristics of human traumatic brain injury such as edema, hemorrhage, contusion, altered cerebral metabolism and inflammation [28], [29]. Clinical traumatic brain injury is broadly categorized as blunt or penetrating. The majority of TBIs are blunt injuries, in which there is a direct impact to the skull without penetration of the intracranial space. The leading causes of blunt injury are falls for the age groups 0–14 and those over 35, while motor vehicle accidents are the leading cause for those between the ages of 14 and 35 [5]. Penetrating injuries result from mechanisms of injury such as gunshot wounds and shrapnel, and differ substantially in management and prognosis. Traumatic brain injury has gained significant attention due to its prevalence in recent military conflicts, where an estimated 28% of combat casualties sustain TBI [30], [31].

Non-steroidal anti-inflammatory drugs (NSAIDs) are a promising candidate for controlling the deleterious effects of inflammation after TBI. Post injury inflammation leads to degradation of the blood brain barrier, edema, increased intracranial pressure, metabolic disturbances, activation of microglia and infiltration of peripheral immune cells [32]–[35]. These immune cells produce reactive oxygen species, which are especially damaging to the lipid rich membranes of the nervous system [36], [37]. Injury induced inflammation also leads to several deleterious effects on cerebral blood vessels [38], [39].

NSAIDs possess well-documented analgesic, antipyretic, and anti-inflammatory effects [40]. However, diffuse distribution of NSAIDs throughout the body leads to an array of adverse side effects, thought to be caused by free carboxylic acid groups and blockage of prostaglandin synthesis in the gastrointestinal system [41]. In order to circumvent the adverse side effects associated with NSAIDs and improve bioavailability, various NSAID prodrugs have been developed that mask carboxylic acid groups through the formation of bioreversible bonds [42]–[44]. In the experimental nanoprodrug used in this study, the carboxylic acid functional groups of each ibuprofen molecule are joined to a tetra ethylene glycol (TEG) in an esterification reaction, preventing them from interacting with off target tissue until hydrolytic or enzymatic cleavage and activation. Each TEG binds two ibuprofen to form Ibu_2_TEG.

Ibu_2_TEG was stabilized by hydrophobic interactions with the antioxidant α-tocopherol. α-tocopherol is the most biologically active form of vitamin E and is believed to be the most potent lipid-soluble antioxidant because it is capable of breaking the chain of propagation of free radical mediated lipid peroxidation [45], [46]. Oxidative damage caused by reactive oxygen species (ROS) is believed to be a major feature in the pathophysiology of many neurodegenerative diseases [37], [47]. Various studies suggest that long-term use of NSAIDs may prevent or delay dementia in Alzheimer’s Disease, which is characterized by an increased inflammatory profile in the brain similar to TBI [48]–[51].

The blood brain barrier (BBB) is a tightly regulated interface between the central nervous system and the circulating blood, formed by CD31+ vascular endothelial tissue. The BBB protects the CNS from edema and neurotoxic macromolecules. When the BBB is functioning normally, it also often blocks the delivery of therapeutics that would be used to treat conditions such as neurodegenerative diseases, CNS infections, and brain tumors. However, in TBI the integrity of the BBB is known to be severely compromised at the site of injury [52], [53]. The destruction of the BBB interface can be a direct result of the traumatic injury itself, as well as due to secondary consequences of inflammation- related mechanisms, metabolic disturbances, and astrocyte dysfunction. This permeability may represent a serendipitous opportunity to deliver drugs to the site of injury [54].

The phenomenon of failing vascular barrier activity has been described in oncology literature as the enhanced permeability and retention (EPR) effect [55], [56]. Many rapidly growing solid tumor types exhibit defects in angiogenesis, resulting in the formation of poorly organized and highly permeable blood vessel structures. Although the etiology for the EPR effect is different in TBI than it is in tumor formation, the effect is the same [52], [57], [58]. Thus, we find it appropriate to describe vascular permeability in TBI using the same term. Just as the EPR effect has been shown to permit an increase in chemotherapeutic nanodrug uptake in tumors, it has also been recently shown to allow proteins chaperoned by polybutyl cyanoacrylate to be delivered to injured tissue in TBI using rats [59]. Therefore, we have focused our effort toward developing and validating an innovative nanoprodrug capable of crossing the damaged BBB and delivering drugs to the site of brain injury. Our paper provides a proof of concept that our novel nanoprodrug can accumulate at the site of injury in a mouse model of CCI.

## Methods

### Ethics Statement

All experiments were approved by the Institutional Animal Care and Use Committee (IACUC) at the Cedars-Sinai Medical Center (protocol #2620). All efforts were made to minimize suffering through the use of anesthesia, analgesia, and post-injury care and monitoring.

### Animals

Male 12-week old C57BL/6 wild type mice (strain #000664) were obtained from Jackson Laboratory (Bar Harbor, Maine). On the day of injury, the placebo group and the treatment group had similar weights (26.01±1.18 g v, 25.48±1.64 g, p = 0.42). Mice were anesthetized with inhalation isoflurane (4% to induction, and 2% maintenance), shaved in the region of cortical impact, and secured in a stereotaxic frame. Mice were then subjected to TBI using electromagnetic controlled cortical impact (CCI) [22], [60]. A 2 mm impactor tip struck the left frontotemporal skull at a velocity of 3 m/s reaching a depth of 2 mm. One mouse assigned to the saline group died on impact, and one mouse each in the IP group and IV group were sacrificed before testing due to a failure to recover a righting reflex as a result of the injury. This reflects an expected 10% attrition due to the severity of the injury delivered.

Eight mice (n = 8) were randomized to placebo group, undergoing CCI and then an intraperitoneal injection of phosphate buffered saline (PBS) after injury. Twelve mice (n = 12) were assigned to the intraperitoneal (IP) treatment group and were given an immediate IP injection of nanoprodrug (100 µl, 0.2 mg/mouse). Six mice (n = 6) were assigned to the intravenous (IV) group, recovered from anesthesia for five minutes, and injected with nanoprodrug (100 µl, 0.2 mg/mouse) via tail vein. All mice were recovered on a warming pad until ambulatory, returned to their cages, and housed in groups of two with a 14∶10 hour light-dark cycle with water and softened chow available *ad libitum*.

### Preparation of NSAID Nanoprodrug

**Figure 1:**
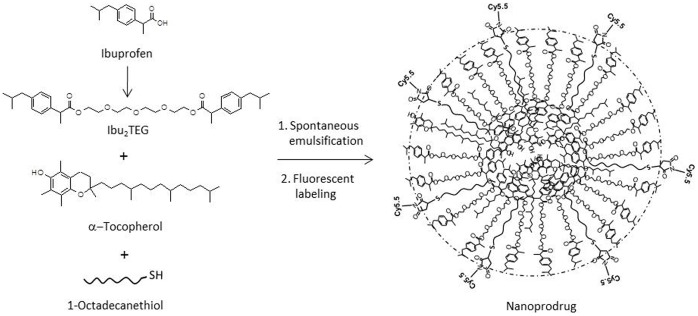
Nanoprodrug preparation and characterization. This chemical schematic shows the molecular structures of the individual ibuprofen molecule, the Ibu_2_TEG complex consisting of two ibuprofen molecules jointed by a tetraethylene glycol (TEG) spacer, the anti oxidant α-tocopherol, and the hydrophobic 1-octadecanethiol which is joined to Cy5.5 after emulsification. The final product is represented on the right hand side of the schematic.

The nanoprodrug is constructed of ibuprofen molecules joined by a tetra ethylene glycol (TEG) spacer in an emulsion with the antioxidant α-tocopherol and 1-octadecanethiol which is irreversibly bonded to the Cy5.5 fluorescent tracer. The combination of two ibuprofen molecules joined by the TEG spacer is referred to as Ibu_2_TEG (Figure 1). The ester bond between each ibuprofen and the TEG spacer is biodegradable, ensuring that the prodrug molecules break down hydrolytically or enzymatically. In contrast, the thioether bond between the Cy5.5 maleimide fluorescent tracer and 1-octadecanethiol is not biodegradable. 1-Octadecanethiol is a water-insoluble sulfur compound with an 18 carbon alkyl chain, which forms a strong hydrophobic assembly with Ibu_2_TEG and α-tocopherol. The nanoprodrug was noted to be highly stable. We incubated the complete nanoprodrug for 48 hours at physiological pH in PBS and did not detect any detachment of Cy5.5 from the nanoprodrug particles (data not shown).

The details of Ibu_2_TEG synthesis are previously described [61]. The nanoprodrug was prepared by the spontaneous emulsification of 50 mg of Ibu_2_TEG, 10 mg α-tocopherol, and 2 mg of 1-octadecanethiol all dissolved in acetone (5 ml) containing polysorbate 80 (0.1% w/v). The organic solution was poured under moderate stirring on a magnetic plate into an aqueous phase prepared by dissolving 25 mg of Pluronic F68 in 10 ml distilled water (0.25% w/v). Following 15 min of stirring, acetone was removed under reduced pressure.. To 2 mL of the thiolated nanoprodrug suspension, 500 µL of 10×PBS and molar equivalent of Cy5.5 maleimide (GE Healthcare) were added. The reaction mixture was incubated overnight at room temperature under light protection. To remove unbound Cy5.5 maleimide, the suspension was purified on a G-25 Sephadex column (GE Healthcare) equilibrated with 20 mM sodium citrate buffer with 0.15 M NaCl. The suspension was dialyzed in a cellulose membrane tube (Sigma #D9777) overnight in distilled water and filtered consecutively through 0.8, 0.45, and 0.2 µm hydrophilic syringe filters (Corning) and stored at 4°C. The concentration of the bound Cy5.5 was determined by mixing 200 µL of nanoprodrug suspension with 800 µL acetonitrile before measuring the optical light absorbance at 675 nm. The concentration was calculated using a standard curve generated with Cy5.5 maleimide.

### Behavioral Testing

Behavioral testing was determined using the Barnes Maze for cognitive function, and the open field and rotorod tests of motor function. The Barnes Maze assessed spatial reference and working memory retention. Ten animals were tested in the Barnes Maze (n = 6 IP, n = 4 placebo). Prior to injury animals received five days of training to locate and enter a hide box within a two-minute time limit. Injury occurred on day 6, and memory retention of the task was assessed on day 7. On day 8, a probe test was conducted as a control, in which the box was moved to a new location to determine if the animals were not using non-memory associated cues (such as the scent of the box) to locate the hide box.

The open field and rotorod tests were conducted twenty-four hours after TBI to assess gross motor function. In the open field test, mice were placed in a plexiglass box, with motion monitored by lasers over the course of one hour. Ambulation is defined as more than two consecutive laser beam breaks. The rotarod test assesses coordination and strength by measuring the time the animal can balance on a rod rotating at constantly increasing angular velocity.

### Fluorescence Imaging

Post mortem brain tissue was imaged using Xenogen 200 Imaging System (Caliper Life Sciences) to localize accumulation of the fluorescent nanoprodrug within the brain. Intact whole brains were imaged and then sectioned for repeat imaging. Frozen tissue was mounted in OCT compound, cryosectioned using a cryotomb (10µm), and stained with hematoxylin and eosin. For fluorescent confocal microscopy, brains were cryosectioned (10µm) and mounted and coverslipped with one drop of mounting medium with DAPI (Prolong Gold, Invitrogen). A fluorescent microscope (Model Upright Zeiss) and a confocal laser-scanning microscope (Leica Microsystem SP5) equipped with a digital camera were used for microscopic analysis.

### Tissue Collection

After behavioral testing and imaging procedures, mice were sacrificed three days after injury using carbon dioxide inhalation followed by cervical dislocation. Brains were then immediately harvested by peeling the skull away and extracting the whole brain onto dry ice for snap freezing. Tissues were stored at −80 degrees Celsius until processing.

### Statistics

Groups are described as means with standard deviations and compared using a two tailed Student’s t-test, with a level of p = 0.05 considered significant.

## Results

### Imaging

**Figure 2:**
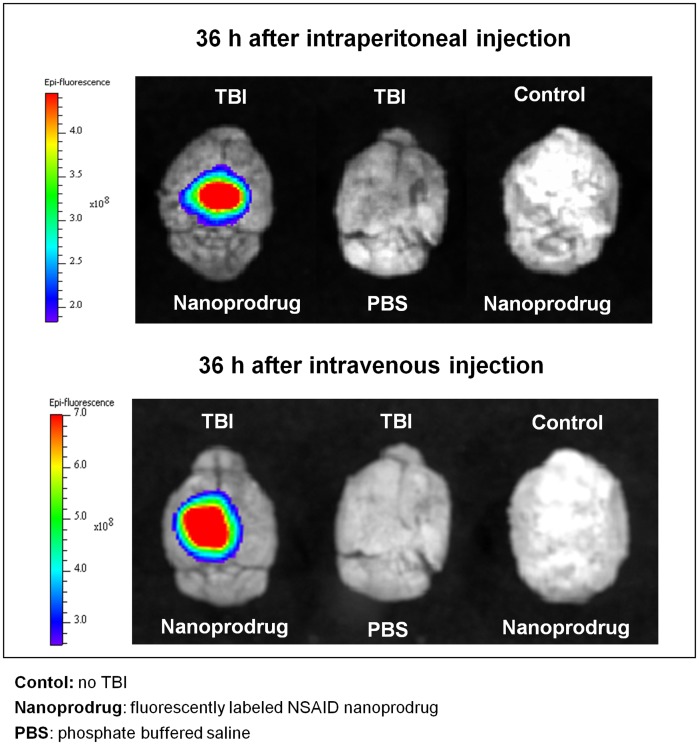
Comparing accumulation for IV and IP administration. The injection of nanoprodrug either IV or IP results in similar accumulation in animals with TBI, while normal animals given nanoprodrug and TBI animals do not show any background flourescence. Brains are oriented with the rostral portion toward the top of the image.

Whole brains were collected from mice 36 hours after injury and nanoprodrug administration. Using Xenogen bioluminescence imaging, the Cy5.5 fluorescent marker was detected at the site of injury on the left parietal region of whole brain (Figure 2). Fluorescence was not detected in uninjured sham animals receiving the nanoprodrug, nor was it detected in TBI animals treated with PBS. Comparing animals treated with IP injection of the drug (Figure 2, upper panel) to animals treated with IV injection of the drug (lower panel), accumulation is similar.

**Figure 3:**
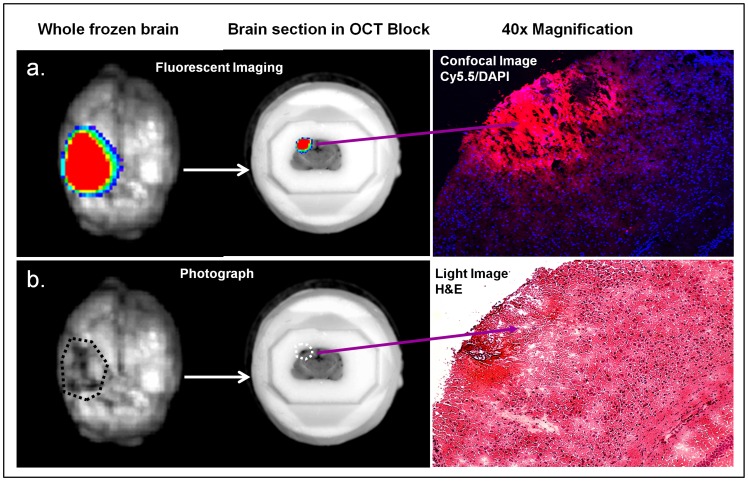
Drug accumulation in the area of injury. Accumulation of the drug in the left parietal area is visualized (a) using fluorescent imaging in the top panels and (b) by traditional photography and hemotoxylin and eosin staining in the lower pannels.

Confocal microscopy of sectioned brains revealed accumulation of the nanoprodrug (pink, Cy5.5) at the area of injury (Figure 3, right upper panel). Nuclei are stained with DAPI nuclear stain in blue. Whole brain photographs exhibit hematoma formation and hemotoxylin and eosin stain of brain tissue slices demonstrates significant tissue disruption of the cortical tissue (Figure 3, lower right panel).

**Figure 4:**
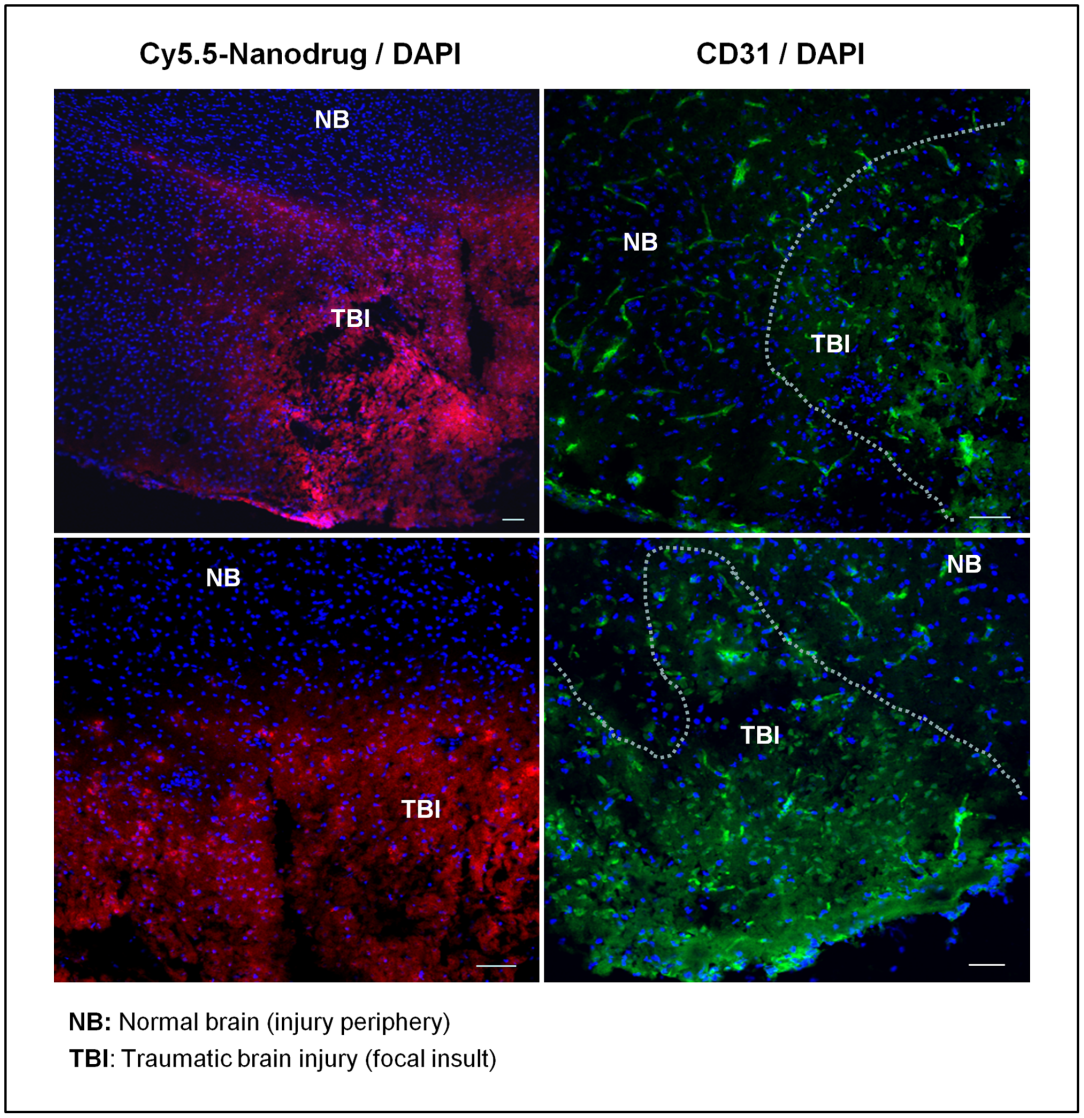
Disorganized vascular structures at the region of nanoprodrug uptake. Representative images from two brains showing nanoprodrug uptake on the left column and CD31 staining of vascular endothelial cells on the right. Outside of the TBI region, vascular structures exist in normal tubular arrangements, but these are disorganized within the region of injury. The nuclei are stained with DAPI are displayed in blue. *Scale bar,* 50 *µ*m.

To investigate the cerebral vasculature, we stained for CD31+ vascular endothelial cells (Figure 4). The tubular structures of vessels are visible outside of the focal region of impact. In contrast, vessel organization is highly disorganized at the area of TBI. This disorganization is highly correlated with regions of increased nanoprodrug uptake.

### Behavior

**Figure 5:**
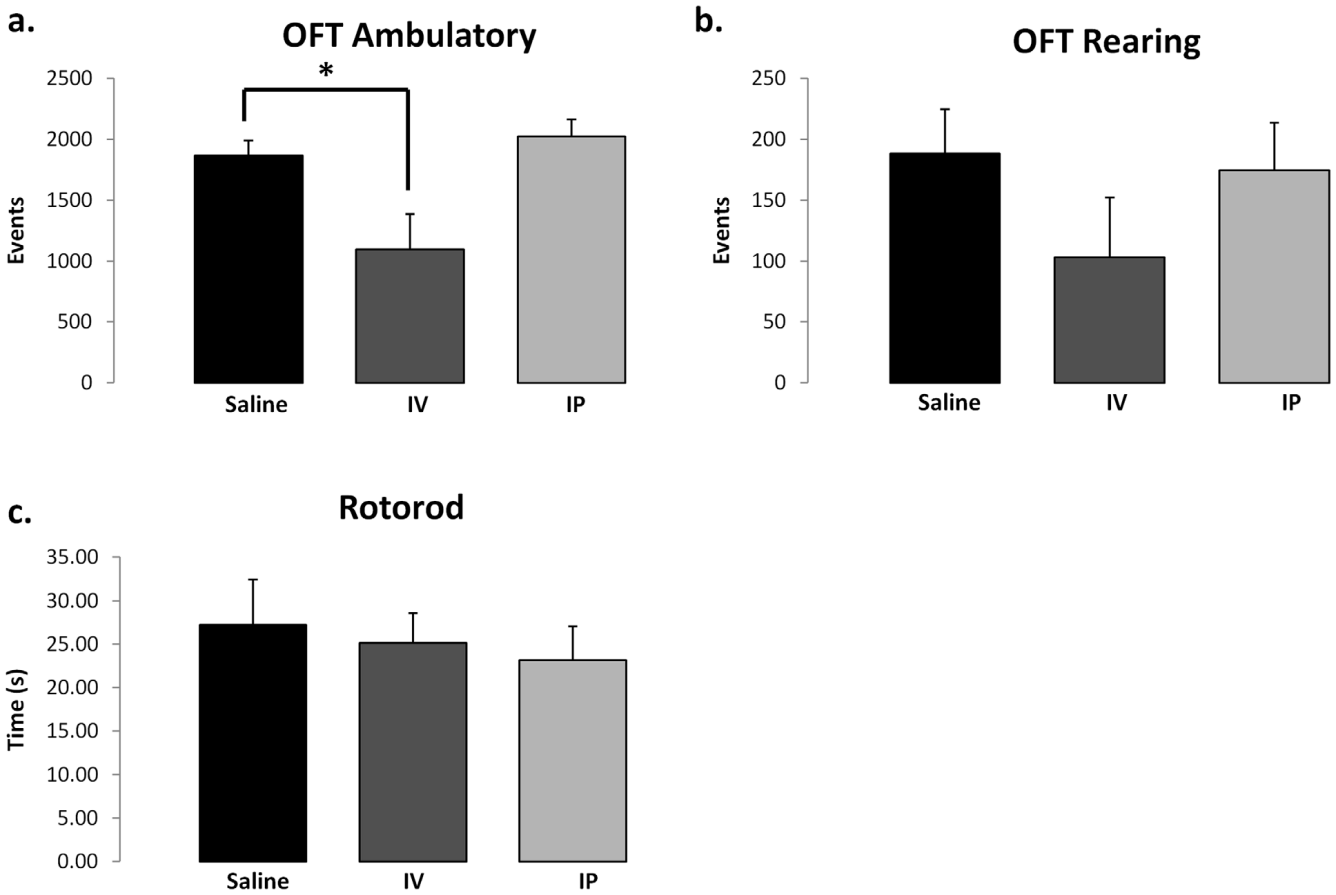
Behavioral testing of motor function using Open Field Test and Rotorod. (a) The number of ambulatory movements over the course of one hour in the Open Field Test (OFT) was reduced for the mice in the IV group. (b) The number of rearing movements in the OFT was not significantly different between groups. (c) Rotorod performance demonstrates that all mice were able to balance on a rotating rod for similar amounts of time. Statistical comparison was performed using a two tailed Student’s t-test, with a level of p = 0.05 considered significant.

**Figure 6:**
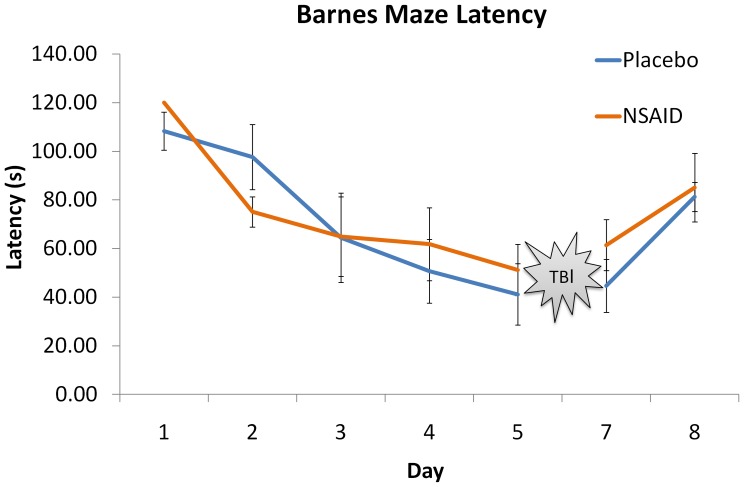
Behavioral testing of memory function using the Barnes Maze. The Barnes Maze was conducted for five days of training followed by traumatic brain injury (TBI). One day after traumatic brain injury (Day 7 on graph), mice did not demonstrate any significant differences in time to find the escape box. Statistical comparison was performed using a two tailed Student’s t-test, with a level of p = 0.05 considered significant. Day 8 serves as a control in which the location of the box is changed to show that mice are not using other cues such as scent to locate the box.

On the open field test, mice treated with the nanoprodrug IP had no significant reduction in ambulation events compared to placebo (nanoprodrug IP 2024±484 v. placebo 1865±302, p = 0.47) or rearing (nanoprodrug IP 174±136 v. placebo 188±90, p = 0.82) (Figure 5). In contrast, mice treated with the nanoprodrug IV had significantly reduced ambulation compared to the control group (nanoprodrug IV 1098±641 v. NS 1865±302, p = 0.005). No difference in rearing events were noted with IV injection of the prodrug (nanoprodrug IV 103±110.6 v. placebo 188±89.5, p = 0.31). No significant differences were noted in rotorod times between groups (nanoprodrug IV 25.1±7.7 s v. placebo 27.2±12.8 s, p = 0.76; nanoprodrug IP 23.2±13.4 s v. placebo 27.2±12.8 s, p = 0.55). No significant differences were noted in latency to find the hide box between groups on post injury day 1 (IP 61.4±10.4 s vs placebo 44.67±11.0 s p = 0.32) (Figure 6). No significant differences were noted between groups on the reversal test (IP 85.06±14.1 v placebo 81.25±6.0 p = 0.84).

## Discussion

Each year, approximately 1.5 million Americans sustain a traumatic brain injury (TBI), resulting in over 50,000 deaths and 80,000 long-term disabilities [5]. The inflammatory response cascade after traumatic brain injury contributes to secondary damage and degeneration as well as post injury repair [38], [53], [62], [63]. Because the cyclooxygenase (COX) system is ubiquitously expressed in the body, previous attempts to treat TBI with COX inhibitors may have been confounded by off target effects. Such side effects are particularly dangerous to trauma patients with TBI because of their increased risk for stress ulcer formation and subsequent GI hemorrhage [64]. In this study, we used a recently developed hydrophobic derivative of the non-selective COX-1 and 2 inhibitor ibuprofen, combined with the antioxidant α-tocopherol and 1-octadecanethiol which binds the Cy5.5 fluorescent tracer. Each pair of ibuprofen molecules is joined by tetra ethylene glycol (TEG), forming Ibu_2_TEG. In Ibu_2_TEG, the carboxylic acid functional groups of the ibuprofens are esterified upon joining to TEG. Not only does this protect the ibuprofen from premature degradation, but it may protect off target tissues from irritation by the acidic carboxylic acid groups. At the region of injury, where the blood brain barrier has increased permeability, we found significant accumulation of the nanoprodrug. Our finding has significant clinical implications as a potential treatment for TBI that is both safe and effective.

**Figure 7:**
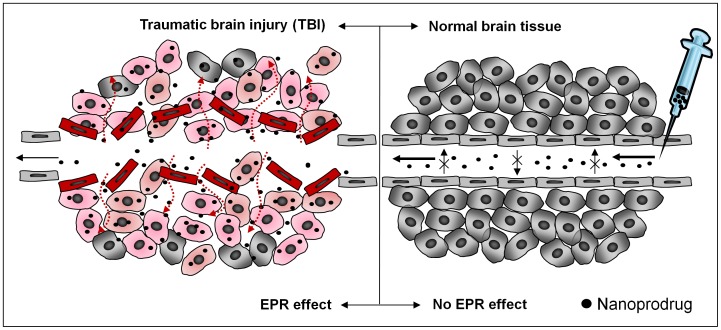
Enhanced Permeability and Retention (EPR) at the site of TBI. This schematic illustrates the difference between healthy brain and injured brain in terms of the structural organization of the blood vessels and how this influences drug delivery. In normal tissue, the blood brain barrier is intact and the nanoprodrug does not penetrate into the tissue. An injured vessel becomes leaky, and the disruption of the blood brain barrier allows for uptake and accumulation of the nanoprodrug particles.

The enhanced permeability and retention (EPR) effect was first described in reference to rapidly growing solid tumors, which often exhibit disorganized angiogenesis [55], [56]. The disorganized vasculature is more permeable and has been reliably shown to lead to increased nanprodrug uptake [61], [65]. The nanoprodrug used in this study was recently demonstrated to be effective in suppressing proliferation of glioma cells [61], [65]. Vascular permeability is disrupted in traumatic brain injury as well, either as a direct result of injury or as a secondary effect of inflammation [52], [57], [58]. Ultimately, this leads to a similar EPR effect, and a similar opportunity to deliver therapeutics (Figure 7). We show disorganized vascular structure at the region of injury by staining CD31+ cells to identify vascular endothelial cells. The normally tubular structures seen in normal brain are replaced by dispersed flourescent signal in injured brain. Regions showing vascular disorganization were well correlated with regions of nanoprodrug accumulation.

**Figure 8:**
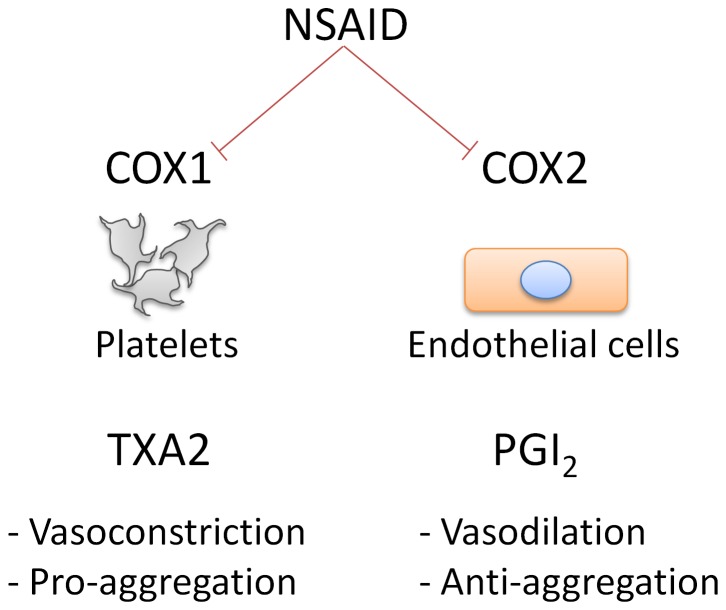
The COX system regulates blood flow and platelet activity. The COX1 enzyme acts in platelets to activate thromboxane A2, which leads to vasoconstriction and enhanced platelet aggregation. The COX2 enzyme acts in endothelial cells to stimulate vasorelaxation and platelet inhibition.

NSAIDs have widely known analgesic, antipyretic, and anti-inflammatory effects. Their mechanism of effect is through COX inhibition. COX enzymes are produced as two isoforms, COX-1 and COX-2. In endothelial tissue, the constitutive production of COX-2 leads to the production of PGI_2_, which causes vasorelaxation and inhibits platelet aggregation. Normal hemostasis is maintained by a balance between this epithelial effect and a COX-1 catalyzed thromboxane A2 activity in platelets, which mediates a vasoconstrictive and pro-aggregation effect [66] (Figure 8). Therefore, our use of a non-selective COX inhibitor would be expected to better maintain a natural hemostatic balance.

In the brain, COX-2 induction is known to be upregulated after traumatic brain injury in rats starting at 3 hours and lasting for at least 12 days [67]. Such elevated production of COX-2 is thought to increase cellular damage, vascular dysfunction, and alterations in cellular metabolism [68]. COX-2 catalyzed production of prostaglandin PGE_2_ results in the production of free radicals. Free radical-induced lipid peroxidation is responsible for massive neuronal death following primary mechanical injury [69], and PGE_2_ itself is also neurotoxic [70], [71]. In the short term, vascular permeability in response to inflammatory cell signaling leads to edema and intracranial hypertension, which further contributes to cell death [63], [72]. In the long term, inflammation has been linked with the development of numerous neurodegenerative diseases including amyotrophic lateral sclerosis, multiple sclerosis, Alzheimer’s Disease, and Parkinson’s Disease [9], [49], [73], [74].

The nanoprodrug contains α-tocopherol (vitamin E) as an antioxidant component and stabilizing structural component. Increasing evidence suggests that vitamin E may play a promising role in the prevention and treatment of oxidative damage-related neurodegenerative diseases [37], [47], [75], [76]. However, its extreme insolubility in water poses a serious limitation to distribution in the aqueous biological environment, limiting its usefulness as a therapeutic intervention. Efforts to make α-tocopherol more water soluble by replacing the lipophilic phytyl chain with more hydrophilic moieties interfere with its antioxidant capabilities and may incur unexpected adverse biological effects [46], [77]. Thus, unmodified α-tocopherol was used in the formulation of the nanoprodrug as a stabilizing and size reducing structural component, in addition to its antioxidant benefits. Despite the hydrophobicity of Ibu_2_TEG and α-tocopherol alone, formation of the two into a nanoparticle generates a large surface area for hydrolytic esterase enzymes to interact and degrade prodrugs, releasing ibuprofen from the surface [61].

### Conclusion

Bioluminescence imaging reveals that the novel NSAID nanoprodrug accumulates at the area of injury, possibly due to an enhanced permeability and retention (EPR) effect. The nanoprodrug targets the injury with high specificity, which may potentially reduce off target effects on other organs. Behavioral testing indicates that only animals receiving the drug intravenously demonstrated a significant reduction in ambulation, whereas animals receiving the drug IP showed equivalent motor function to animals receiving PBS control. Rotorod and Barnes Maze showed no significant differences in outcome with the use of the NSAID nanoprodrug.

We believe this study demonstrates the feasibility of using the NSAID nanoprodrug to target TBI. Further testing will explore off target organ toxicities, nanoprodrug half-life, and determine the minimal effective dose. Further preclinical studies may investigate the use of the nanoprodrug in a blast injury model in addition to controlled cortical impact. The novel combination of the nanoprodrug delivery strategy and NSAID therapy represents a promising therapeutic modality for the treatment of many types of TBI.
